# ‘Never waste a crisis’: a commentary on the COVID‐19 pandemic as a driver for innovation in maternity care

**DOI:** 10.1111/1471-0528.16996

**Published:** 2021-11-23

**Authors:** LMM van den Berg, G Thomson, A de Jonge, M‐C Balaam, G Moncrieff, A Topalidou, S Downe, the ASPIRE COVID‐19 Team, George Ellison, Alan Fenton, Alexander Heazell, Carol Kingdon, Zoe Matthews, Alexandra Severns, Alison Wright, Naseerah Akooji, Jo Cull, Nicola Crossland, Claire Feeley, Beata Franso, Steph Heys, Rebecca Nowland, Arni Sarian, Maria Booker, Jane Sandall, Jim Thornton, Tisian Lynskey‐Wilkie, Vanessa Wilson, Rebecca Abe, Tinuke Awe, Toyin Adeyinka, Ruth Bender‐Atik, Lia Brigante, Rebecca Brione, Franka Cadée, Elizabeth Duff, Tim Draycott, Duncan Fisher, Annie Francis, Arie Franx, Lucy Frith, Louise Griew, Clea Harmer, Caroline Homer, Marian Knight, Amali Lokugamage, Amanda Mansfield, Neil Marlow, Trixie Mcaree, David Monteith, Keith Reed, Yana Richens, Lucia Rocca‐Ihenacho, Mary Ross‐Davie, Seana Talbot, Myles Taylor, Maureen Treadwell

**Affiliations:** ^1^ Department of Midwifery Science AVAG/Amsterdam Public Health Amsterdam University Medical Centre Vrije Universiteit Amsterdam Amsterdam The Netherlands; ^2^ School of Community Health & Midwifery Faculty of Health and Care University of Central Lancashire Preston UK

The coronavirus (COVID‐19) pandemic has resulted in rapid changes in many areas of healthcare worldwide.^1^ Some organisational and governance controls on innovation have been relaxed, to enable rapid adaptation to changing circumstances. The speed of innovation raises a range of ethical, governance and organisational issues. It is important to assess what changes have been instituted, which ones should be maintained, and how to encourage effective innovations in future. Maternity care provides an exemplar case within the broader healthcare setting, given the imperative to provide both safe and personalised care for optimal outcomes. Some pandemic‐related changes in maternity services, such as restricting women's opportunities for companionship during ultrasound scans or throughout labour, or limiting parental visiting to neonatal units, have been associated with psychological harm.^2^ Other changes provide more positive impacts, including reports of more individualised and efficient care associated with the increased use of telemedicine.^3^ We undertook a documentary analysis of national policy and service‐user organisation responses to the pandemic in the United Kingdom (UK) and the Netherlands (NL), as part of the Achieving Safe and Personalised maternity care In Response to Epidemics (ASPIRE COVID‐19) study. The overall aim of ASPIRE COVID‐19 is to identify ‘what works’ in providing maternity care during the current and future pandemics, or similar health crises. The NL was chosen as the comparator for the UK because there were known differences in the organisation of maternity services during the COVID‐19 pandemic between the two countries, especially for place of birth. Here we report on activities described as new or expanded innovations in 290 documents produced by 17 key professional and service‐user organisations in the NL and the UK between February and December 2020 (Table 1). We included strategic papers, guidelines, protocols and updates for healthcare professionals, such as newsletters.

**Table 1:** Professional and service‐user organisations (*n* = number of collected documents)

	United Kingdom	Netherlands
Professional organisations	Royal College of Midwives (RCM) (*n* = 47) Royal College of Obstetricians and Gynaecologists (RCOG) (*n* = 32) Society of Radiographers (SoR) (*n* = 12) Royal College of Paediatrics and Child Health (RCPCH) (*n* = 6) Mothers and Babies: Reducing Risk through Audits and Confidential Enquiries across the UK (MBRRACE‐UK) (*n* = 1) National Health Service England/Scotland/Wales/Northern Ireland (NHS) (*n* = 21)	Royal Dutch Organisation of Midwives (KNOV) (*n* = 62) Dutch Association for Paediatrics (NVK) (*n* = 6) Dutch Society for Obstetrics and Gynaecology (NVOG) (*n* = 16) College of Perinatal Care (CPZ) (*n* = 19) Knowledge Centre for Maternity Care Assistants (KCKZ) (*n* = 18) Professional Organisation of Dutch Sonographers (BEN) (*n* = 4)
Service‐user organisations	Association for Improvements in the Maternity Services (AIMS) (*n* = 9) Birthrights (BR) (*n* = 27) Still Birth and Neonatal Death charity (SANDS) (*n* = 4) Birth Companions (BC) (*n* = 3)	Birth movement (BM) (*n* = 3)

## The nature of the innovations

In both countries, some innovations emerged in response to the pandemic, and some that were already in place gained momentum, such as telehealth and early discharge following birth. The innovations and expanded practices described in the included documents are summarised in Table 2, organised by topic. We then discuss the type of innovations and practices that were reported, and the potential ethical, governance and organisational implications of rapid innovation in maternity care specifically, and for healthcare in general.

**Table 2:** New innovations and expanded practices in NL and UK maternity care as reported in documents produced by national organisations during the COVID‐19 pandemic

Innovation category	Mentioned innovation	NL	UK
Telehealth	Telephone appointments (prenatal and postnatal)	X	X
	Video appointments (prenatal and postnatal), which needed other innovations:	X	X
	Technology so that healthcare providers can perform digital consultations securely (e.g. Mobilea)	X	
	Training for maternity care staff on the provision of remote antenatal and postnatal consultations	X	X
	Video calling partner/other preferred person for women during appointments attended alone	X	X
	E‐Health:		
	Blood pressure monitoring at home	X	X
	Glucose monitoring at home		X
	Urine monitoring at home		X
	More focus on digital information and education for pregnant women, e.g. via a Q&A with care providers, online information videos, and online communities	X	X
	Online Centering Pregnancy and antenatal classes	X	X
	Use of headphones during birth to hear the voice of the private doula	X	
	Telephone or video call helplines, or email for urgent enquiries from pregnant women, to be reviewed and responded to by maternity care staff		X
	Video tours of hospitals	X	X
	Provision of a (short) film of ultrasound scans for women to give to their partners	X	X
	Increased use of social media and local charities in the dissemination of important information, such as positive social media narratives.	X	X
Digital communication	Improved digital sharing of patient information between care providers, such as	X	X
	Use of electronic record systems	X	X
	Use of phone, computer or web‐based applications to share patient data easily and securely	X	
	Improved digital communication between different care providers: through meetings, webinars, online conferences and education courses	X	X
Staff wellbeing	Psychological support to improve staff wellbeing	X	X
	More frequent and improved rest and break facilities (such as more comfortable seating)		X
	Additional practical support, e.g. availability of childcare facilities and parking spaces		X
	Shorter working shifts	X	X
A resilient maternity care system	Escalation plans in case of major capacity problems	X	X
	Digital storage of important work documents to run a midwifery practice/hospital department, in case of major capacity problems	X	X
	Crisis app groups on phones to connect different disciplines in maternity care quickly in an emergency (organisational emergencies, not patient emergencies)	X	
	Development of novel ways to transfer women in non‐urgent situations during labour (e.g. by dedicated taxis), for potential ambulance capacity issues		X
	Novel locations for birth, such as such the use of hotels as birth centres		X
Strengthening community provision	Altered methods for induction of labour, enabling women to be at home during early labour.		X
	Commissioning of new off‐hospital locations, e.g. ultrasound scans in new community locations		X
	Increased individualisation in the schedule of antenatal appointments (e.g. fewer appointments where that is the woman's preference)	X	X
	Provision of access to midwifery support at home in early labour, enabling women to remain at home for longer		X
	Establishment of new continuity models of care		X
	Early discharge from hospital		X
	Expansion of the role of the primary care midwife, so that secondary care is less likely to be necessary	X	
	Attention for financial support of parents (to be) (e.g. active providing of financial information)		X

### Telehealth

There is significant interest in telehealth/remote health care as a result of the pandemic.^3^ Our data documents the general move, in both countries, to shift in‐person appointments to virtual appointments where possible and appropriate. The pandemic has hastened digital developments within healthcare. Some prenatal and postnatal appointments became remote in both countries, particularly where no physical examinations were required and there was no known indication for face‐to‐face input. Innovations were then implemented to make digitisation of care possible, including different ways to provide secure remote care. The NL documents provided relevant detail, such as examples of online and telephone applications that meet the safety standards required by Dutch law. In the UK, healthcare documents placed more emphasis on advice around the appropriate use of telemedicine by staff, for example to use a neutral background in a video conversation.

As the pandemic progressed, more attention was placed on the advantages and disadvantages of remote care, with the aim of optimising the benefits and reducing any risks. Various organisations, including the Royal Dutch Organisation of Midwives (KNOV), Dutch Association for Paediatrics (NVK), Royal College of Obstetricians and Gynaecologists (RCOG) and Royal College of Midwives (RCM), emphasised the benefits of using video consultations over telephone appointments. Video consultation then became the preferred method for remote appointments. However, there was heightened awareness among service user organisations and professional organisations such as Birth Companions (BC) and RCOG that remote care was not suitable for all, and that certain groups of women could be disproportionately disadvantaged due to digital poverty, lack of privacy at home or language restrictions. Organisations within both countries identified safeguarding concerns in relation to the difficulties associated with identifying domestic violence and/or mental health issues, where remote technology is used.

Digital information innovations for parents were stimulated by the need to provide structured, regularly updated and congruent information. In both countries there was an emphasis in policy documents on the use of social media, and on helplines and online groups for service users. Information for parents was provided through the digital platforms available to healthcare organisations and videos on YouTube (for example, videos on breastfeeding and how to bath a baby were produced by maternity care assistant organisations in the NL) and hospital tours were provided online.

### Digital communication

The pandemic boosted developments around the digitisation and digital exchange of patient data. In the UK, the emphasis was on the increased use of electronic patient records. In the NL these developments had already occurred, and the concern was how to share electronic patient records across healthcare providers, leading to the implementation of phone (e.g. Siilo), computer, (e.g. BabyConnect) and web‐based applications (e.g. CareCodex Foundation).

Several organisations from both countries, including KNOV, NVOG and RCOG, highlighted the opportunities for organising remote activities for healthcare providers. These included digital meetings which could be both national and international, webinars, education courses and online conferences.

### Staff wellbeing

Innovations to improve staff wellbeing were implemented in both countries in response to reports of high levels of exhaustion and stress among maternity care providers. In the NL, the KNOV COVID‐19 taskforce stated that, although staff had initially been stimulated by the unique and pressing nature of the situation, as the crisis progressed they had become fatigued. Organisations in both countries produced manuals and strategies to prevent stress and to stimulate staff wellbeing. These included ‘wobble rooms’ where staff could relax if they were feeling they could not cope during a shift, and broader policies, such as not to message colleagues after 18.00 hours.

### A resilient maternity care system

The COVID‐19 pandemic exposed vulnerabilities in both countries' maternity care systems, particularly in terms of hospital and staff capacities. Both countries introduced innovations to help mitigate these vulnerabilities and to develop a more resilient maternity care system. These included more community‐based birth locations (e.g. the use of hotels as birth centres), the development of escalation plans (illustrating the actions that need to be taken when there is a crisis that has an impact upon capacity) and the digital storage of key documents. WhatsApp groups for communication were set up among maternity care providers, and alternative transport options (e.g. dedicated taxis) were put in place for non‐emergency transport during birth in case of shortages in ambulance provision.

### Strengthening community provision

Further innovation strengthened the provision of both primary and community care. The aim of these innovations was primarily to reduce the volume of patients within hospitals in order to minimise capacity problems and infection risk for both service users and staff. Examples included the increased use of methods to enable early labour at home after induction of labour, unless there were complications requiring hospital attendance. Women booked for hospital birth were also advised to remain at home during early labour. In the UK, the pre COVID‐19 drive for continuity of carer (CoC) continued, although some CoC projects were temporarily discontinued. Some hospital Trusts established new CoC provision during the pandemic.

## Comment

The documents we reviewed highlight many innovations in maternity care resulting from the COVID‐19 pandemic. Some were completely new; others had previously been used but were rolled out rapidly as the need arose. In addition to the more familiar technical innovations (e.g. telemedicine), we identified innovations relating to staff wellbeing, resulting in a more resilient maternity care system and in strengthening community care.

The innovations which were implemented during COVID‐19, largely to minimise infection risk, may be continued post‐pandemic, due to the benefits that have been observed for organisations, staff and service‐users. The benefits of fully embracing pre‐existing guidelines also became apparent, according to national documents. For example, there was increased encouragement of women to remain at home during early labour, with the support of a midwife, to reduce the impact on hospital capacity as well as minimising infection risk. This guidance was in place pre‐pandemic because women who remain at home during early labour have a lower risk of intervention, and support at home during this phase of labour increases maternal satisfaction.^4^, ^5^

A resilient maternity care system requires the resources and capacity to cope with large‐scale stressful events, such as natural disasters and pandemics.^6^ Some of the innovations we identified are directly linked to short‐term challenges, such as the development of escalation plans and digital storage of key documents needed to run a midwifery practice and/or hospital department, in case of major capacity problems. The pandemic also highlighted long‐term challenges, such as strengthening the workforce.^7^ This was particularly apparent with the increased risk of anxiety, stress and burnout as a result of working during COVID‐19. These conditions decrease staff quality of life and increase the potential for staff to leave the profession or take early retirement.^8^ During the pandemic, attempts were made to limit the risk of emotional exhaustion and to increase staff wellbeing, via better rest and break facilities, as well as psychological support. The continuation of these psychological support mechanisms may contribute to a more positive work environment, to lower staff attrition rates, and to improve outcomes for women, birthing people, infants, and families.

All of these innovations (including the roll out of existing practices) have potential ethical, governance and organisational implications for maternity care specifically and for healthcare in general. In contrast to the idea of careful development and testing of new practices for efficacy, effectiveness, feasibility, acceptability and equity, there was no time for evaluation, and many new practices were implemented almost overnight as solutions and work‐arounds to emerging problems.^9^ This has resulted in a dynamic and creative space that has generated or catalysed valuable new approaches to healthcare. Some of these changes (such as support for staff wellbeing) are self‐evidently beneficial. However, the rapid introduction of others (such as telehealth) raises the potential for over‐extension of techniques that might not work for all, might not be affordable in the longer‐term, could disadvantage some and that might have longer term adverse effects. It is critical to invest time and resources to find out what works, for whom, in what circumstances and why, and to ensure that the use of new approaches is equitable, acceptable and feasible.^9^ There are some studies addressing this issue, particularly in relation to telehealth.^10^ Extension of such analyses to other areas of innovation is especially important in a context where pandemic innovations are already becoming normalised in practice, as de‐implementation can be more challenging than implementation.^11^ The time has come to learn which innovations worked best from a service‐user, professional and organisational perspective, and to use this knowledge to build infrastructure and practices to enable resilient and sustainable maternity care systems, both for a post‐COVID‐19 future, and in anticipation of any future healthcare crisis.

### Disclosure of interests

None declared. Completed disclosure of interests forms are available to view online as supporting information.

### Contribution to authorship

Conceptualisation: GT, AJ, AT, SD. Methodology: LMMB, GT, AJ, M‐CB, GM, AT, SD. Investigation: LMMB, GT, M‐CB, GM. Formal analysis: LMMB, GT, M‐CB, GM. Data curation: LMMB, GT, AT. Writing—original draft preparation: LMMB. Writing—review and editing: LMMB, GT, AJ, M‐CB, GM, AT, SD. Visualisation: LMMB, AJ, SD. Supervision: GT, AJ, SD. Project administration: GT, AT, SD. Funding acquisition: AT, SD, GT, AJ. All authors have read and agreed to the published version of the manuscript.

### Details of ethics approval

Documents and materials used in this inter‐ and intra‐national policy and public information documentary review are publicly available on Trust, governmental and professional organisation websites, or are available on request. Ethics approval is not required for this.

### Funding

This research is funded by the Economic and Social Research Council (ESRC), as part of UK Research and Innovation's rapid response to COVID‐19 (grant number ES/V004581/1). Full details of the main study are available via ResearchRegistry (researchregistry5911) and via UKRI Gateway (https://gtr.ukri.org/projects?ref=ES%2FV004581%2F1).

## Supporting information

Supplementary Material

Supplementary Material

Supplementary Material

Supplementary Material

Supplementary Material

Supplementary Material

Supplementary Material

## Data Availability

The data that support the findings of this study are available from the corresponding author upon reasonable request.▪

## ASPIRE COVID‐19 Collaborative Group

### Co‐investigators

Soo Downe, University of Central Lancashire; George Ellison, University of Central Lancashire; Alan Fenton, Newcastle upon Tyne Hospitals NHS Foundation Trust; Alexander Heazell, University of Manchester; Ank de Jonge, Amsterdam University Medical Center; Carol Kingdon, University of Central Lancashire; Zoe Matthews, University of Southampton; Alexandra Severns, NHS England and NHS Improvement North West; Gill Thomson, University of Central Lancashire; Anastasia Topalidou, University of Central Lancashire; Alison Wright, Royal Free Teaching Hospital in London.

### Research Staff

Naseerah Akooji, University of Central Lancashire; Marie‐Clare Balaam, University of Central Lancashire; Jo Cull, University of Central Lancashire; Lauri van den Berg, Amsterdam University Medical Center; Nicola Crossland, University of Central Lancashire; Claire Feeley, University of Central Lancashire; Beata Franso, Amsterdam University Medical Center; Steph Heys, University of Central Lancashire; Gill Moncrief, University of Central Lancashire; Rebecca Nowland, University of Central Lancashire; Arni Sarian, University of Central Lancashire.

### Steering committee

Maria Booker, Birthrights; Jane Sandall, Kings College London; Jim Thornton (chair), The University of Nottingham; Tisian Lynskey‐Wilkie, University of Central Lancashire; Vanessa Wilson, East Lancashire Hospitals NHS Trust.

### Stakeholder Group

Rebecca Abe and Tinuke Awe, FivexMore; Toyin Adeyinka, MVP BAME Group; Ruth Bender‐Atik, The Miscarriage Association; Lia Brigante, RCM; Rebecca Brione, Birthrights; Franka Cadée, International Confederation of Midwives (ICM); Elizabeth Duff, Expert; postnatal care; Tim Draycott, Royal College of Obstetricians and Gynaecologists (RCOG); Duncan Fisher, Fathers included/Family included/the Family Initiative; Annie Francis, Neighbourhood Midwives; Arie Franx, Erasmus MC; Lucy Frith, University of Liverpool; Louise Griew, National Maternity Voices; Clea Harmer, SANDS; Caroline Homer, Burnet Institute; Australia; Marian Knight, National Perinatal Epidemiology Unit (NPEU); Amali Lokugamage, University College London; Amanda Mansfield, London Ambulance Service Trust; Neil Marlow, University College London; Trixie Mcaree, NHS England; David Monteith, Grace in Action; Keith Reed, Twins Trust; Yana Richens, UCL & City University; Lucia Rocca‐Ihenacho, Midwifery Unit Network; Mary Ross‐Davie, RCM Scotland; Seana Talbot, BirthWise NI; Myles Taylor, British Maternal and Fetal Medicine Society; Maureen Treadwell, Birth Trauma Association.
